# Polymyxin-B hemoperfusion in septic patients: analysis of a multicenter registry

**DOI:** 10.1186/s13613-016-0178-9

**Published:** 2016-08-08

**Authors:** Salvatore Lucio Cutuli, Antonio Artigas, Roberto Fumagalli, Gianpaola Monti, Vito Marco Ranieri, Claudio Ronco, Massimo Antonelli, The EUPHAS 2 Collaborative Group, Riccardo Maviglia, Sandra Cicconi, Davide Silvestri, Giuseppe Bello, Alessandra Brendolan, Federico Nalesso, Gianluca Villa, Pasquale Piccinni, Erica Martin, Vincenzo Cantaluppi, Sergio Vesconi, Giampaolo Casella, Egidio Fasanella, Michele Debitonto, Gianmario Monza, Angelo Blasetti, Rosaria Coletta, Michele D’Ambrosio, Gilda Cinnella, Patrizia Murino, Eugenio Piscitelli, Gaetano Centonze, Marco Cucurachi, Giuseppe Altieri, Vincenzo Leonardo, Anna Sara Idra, Goffredo del Rosso, Maria Polidoro, Nicola Stigliano, Giuseppe Pittella, Gianluca Paternoster, Giuseppe Pulito, Daniela Puscio, Diego Cingolani, Gabriele Falzetti, Pietro Vecchiarelli, Francesco Giunta, Francesco Forfori, Giacomo Castiglione, Stefano Greco, Carlo Capra, Luciano Crema, Leonor Tamayo, Cristina Urbano, Brunello Pezza, Nadia Zarrillo, Pasquale di Monaco, Giuseppe Climaco, Pasquale de Negri, Pasqualina Modano, Riccardo Pagliarulo, Claudio Petrillo, Tania Stripoli, Roberto Oggioni, Laura Campiglia, Anna Rita Valletta, Manuela Lugano, Domenico Milella, Laura Micucci, Ursula Reist, Rolf Ensner, Christian Gianbarba, Lukas Brander, Rajib Paul, Rajesh Crawla, Sanjeev Jasujia, Rajesh Pande, Pratibha Dileep, Sankaran Sundar, Raju Ganesan, Sandeep Dewan, Vivek Nangia, Raj Kumar Mani, Omender Singh, Pracee Sathe, Gupta Sachin, Pradeep M. D’Costa, Samavedam Srivanas, Yogendra Pal Singh, Kent Doi, Fumika Taki, Ricard Ferrer Roca, Eduardo Romay Medina, Josè Gernacho, Francisco Martí, Alberto Martinez-Ruiz, Fernando Martinez-Sagasti, Rafael Zaragoza Crespo, Paola Torti, Valeria Terzi

**Affiliations:** 1 Department of Intensive Care and Anaesthesiology, Agostino Gemelli University Hospital, Catholic University of the Sacred Heart, Largo A. Gemelli, 8, 00168 Rome, Italy; 2 Critical Care Center, Sabadell Hospital, CIBER Enfermedades Respiratorias, Corporació Sanitària Universitària Parc Taulí, Universitat Autònoma de Barcelona, Sabadell, Spain; 3 Department of Anaesthesia and Intensive Care Medicine, Niguarda Ca’ Granda Hospital, University of Milan-Bicocca, Milan, Italy; 4 Department of Anaesthesia and Intensive Care Medicine, University of Rome “La Sapienza”, Rome, Italy; 5 Department of Nephrology, Dialysis and Transplantation, International Renal Research Institute of Vicenza (IRRIV), San Bortolo Hospital, Vicenza, Italy

**Keywords:** Sepsis, Septic shock, Infection, EAA, Extracorporeal endotoxin removal, Polymyxin-B hemoperfusion

## Abstract

**Background:**

In 2010, the EUPHAS 2 collaborative group created a registry with the purpose of recording data from critically ill patients suffering from severe sepsis and septic shock treated with polymyxin-B hemoperfusion (PMX-HP) for endotoxin removal. The aim of the registry was to verify the application of PMX-HP in the daily clinical practice.

**Methods:**

The EUPHAS 2 registry involved 57 centers between January 2010 and December 2014, collecting retrospective data of 357 patients (297 in Europe and 60 in Asia) suffering from severe sepsis and septic shock caused by proved or suspected infection related to Gram negative bacteria. All patients received atleast one cycle of extracorporeal endotoxin removal by PMX-HP.

**Results:**

Septic shock was diagnosed in 305 (85.4 %) patients. The most common source of infection was abdominal (44.0 %) followed by pulmonary (17.6 %). Gram negative bacteria represented 60.6 % of the pathogens responsible of infection. After 72 h from the first cycle of PMX-HP, some of the SOFA score components significantly improved with respect to baseline: cardiovascular (2.16 ± 1.77 from 3.32 ± 1.29, *p* < 0.0001), respiratory (1.95 ± 0.95 from 2.40 ± 1.06, *p* < 0.001) and renal (1.84 ± 1.77 from 2.23 ± 1.62, *p* = 0.013). Overall 28-day survival rate was 54.5 % (60.4 % in abdominal and 47.5 % in pulmonary infection). Patients with abdominal infection treated with PMX-HP within 24 h from the diagnosis of septic shock had a 28-day survival rate of 64.5 %. Patients showing a significantly cardiovascular improvement after PMX-HP had a 28-survival rate of 75 % in comparison to the 39 % of patients who did not (*p* < 0.001). Cox regression analysis found the variation of cardiovascular, respiratory and coagulation SOFA to be independent covariates for 28-day survival. In European patients were observed a higher 28-day (58.8 vs. 34.5 %, *p* = 0.003), ICU (59 vs. 36.7 %, *p* = 0.006) and hospital survival rate (53.2 vs. 35 %, *p* = 0.02) than in Asian patients. However, the two populations were highly heterogeneous in terms of source of infection and severity scores at admission.

**Conclusion:**

The EUPHAS 2 is the largest registry conducted outside Japan on the clinical use of PMX-HP in septic patients. Data analysis confirmed the feasibility of PMX-HP to treat septic patients in daily clinical practice, showing clinical benefits associated with endotoxin removal without significant adverse events related to the extracorporeal technique.

**Electronic supplementary material:**

The online version of this article (doi:10.1186/s13613-016-0178-9) contains supplementary material, which is available to authorized users.

## Background

Polymyxin-B hemoperfusion (PMX-HP) [1] is a blood purification technique designed to bind and neutralize circulating endotoxins by extracorporeal removal, inhibiting the progression of the septic cascade mediated by lipopolysaccharide (LPS) [2–16], the core lipid portion of the Gram negative bacterial wall. Endotoxin is removed from the blood by extracorporeal hemoperfusion with a polymyxin-B-adsorbing cartridge that binds endotoxin through covalent and ionic bonds. This therapy was first introduced in Japan in 1994 and 10 years later it was introduced in Europe.

The most common indication for PMX-HP has been septic shock due to abdominal infections. In case of impairment of the intestinal mucosa, LPS can translocate into the bloodstream. The consequent huge activation of the immune system can lead to the dysfunction of multiple organs until the loss of their function (MOF). The importance of endotoxin to determine the unfavorable evolution of sepsis related to Gram negative bacteria has been clearly demonstrated [2–15].

Several trials have been carried out to verify the hypothesis that removing circulating endotoxin from the blood of patients suffering from abdominal septic shock could improve their clinical outcome, in terms of organ dysfunction and mortality [4–15].

The Italian nationwide Early Use of Polymyxin B Hemoperfusion in Abdominal Septic Shock (EUPHAS) Randomized Control Trial (RCT) [15], demonstrated a significant improvement in cardiovascular response and outcome of patients treated by PMX-HP for severe sepsis and septic shock due to abdominal infections. On the other hand, the French ABDOMIX RCT [16] failed to demonstrate clinical benefits of PMX-HP application in a cohort of patients suffering from peritonitis-induced septic shock after emergency surgery.

Furthermore, PMX-HP has been applied in septic patients with source of infection different from the abdomen as respiratory system [17].

Due to the conflicting evidence given by EUPHAS and ABDOMIX RCTs in sepsis sustained by abdominal source of infection and new potential indication highlighted by case report on respiratory infections, the real application of PMX-HP in daily clinical practice is unclear.

In 2010, the EUPHAS 2 project [18] was launched, to create an international registry with the purpose of recording data from patients affected by Gram negative related severe sepsis and septic shock treated with PMX-HP. The aim of the project was to evaluate the application of PMX-HP in the daily clinical practice, both in order to verify the reproducibility of data currently available in the literature and to evaluate the population actually elected for the therapy.

The EUPHAS 2 project is divided into two phases. The first retrospective phase aimed to collect data from at least 250 patients. The analysis of the data from this phase was used to optimize the data collection for the second prospective phase, which aims to collect data from a much larger patient population. The results from the second phase will be used to identify subpopulations of patients who may benefit from this treatment more than others.

A preliminary analysis of EUPHAS 2 retrospective phase [19] confirmed the results of EUPHAS RCT in abdominal septic patients. In the current paper are presented the full results from the first retrospective phase of EUPHAS 2.

## Methods

The EUPHAS 2 project was a voluntary registry specifically conceived for the evaluation and analysis of PMX-HP application in the real clinical life.

All the patients admitted to the 57 participating centers with suspected Gram negative related severe sepsis and septic shock unresponsive to standard care [20] were included in the registry. Forty-six centers were located in Europe (Italy, Spain and Switzerland), and eleven centers in Asia (India). Data collection started in January 2010 and ended in December 2014.

The definition of severe sepsis and septic shock followed the consensus guideline enounced by Surviving Sepsis Campaign [20].

The Gram negative etiology of sepsis was suspected in relation to the source of infection (the abdomen is colonized by Gram negative species) or proven by microbiological tests. In 24 out of 57 centers involved in the registry, LPS might be assessed on peripheral blood sample by the neutrophil-dependent chemiluminescence-based endotoxin activity assay (EAA) [24]. EAA quantifies endotoxin levels through a relative scale ranking from 0 to 1. EAA is characterized by a sensitivity of 85.3 %, a specificity of 44 %, a negative predictive value of 98.6 % for the exclusion of Gram negative infection and 94.8 % for the exclusion of all infection [21]. Marshall et al. [21] demonstrated a progressive mortality increase related to endotoxin activity (EA) rising value. Highest mortality rate was observed for EA greater than 0.6, whereby it is considered the threshold to start the LPS removal.

PMX-HP is not ruled by specific guidelines and this therapy is recommended in severe sepsis and septic shock unresponsive to standard care [20], caused by proved or suspected infection related to Gram negative bacteria. All patients received fluid infusion and vasopressor therapy if needed [20] and independently to PMX-HP.

In all patients, PMX-HP was performed using Toraymyxin^®^ cartridge (Toray Medical Company, Tokyo, Japan) connected to the patient through a 10–12 French veno-venous catheter inserted into the right or left femoral or internal jugular vein and the pump flow rate was 80–120 mL/min.

Each session of PMX-HP lasted 2 h. In relation to clinical condition of the patient and EA (where available), the clinician decided to perform a second cycle of PMX-HP with a delay of 24 h from the first treatment.

Heparin intravenous administration as anticoagulant was set starting from a bolus of 3000 UI before the beginning of PMX-HP with a continuous infusion of 20 UI/kg/h during the treatment. The recommended dose of heparin could be modified in relation to the hemorrhagic risk of the patient.

Main data collection included: timing of diagnosis of severe sepsis/septic shock and treatment with PMX-HP; hemodynamic status; septic organ function impairment; outcome at 28 days, ICU and hospital discharge. Septic progressive organ function impairment was monitored with SOFA score [22–25], hemodynamic status was evaluated with mean arterial pressure (MAP), lactates concentration in the blood and vasopressor load expressed as the inotropic core [(dopamine dose x 1) + (dobutamine dose x 1) + (adrenaline dose x 100) + (noradrenaline dose x 100) + (phenylephrine dose x 100), wherein all doses are expressed as μg/kg/min]. SOFA score, inotropic score and lactates concentration in the blood were assessed at the time of the first PMX-HP (*t*_0_) and every 24 h until 72 h (*t*_72_) from *t*_0_.

Patients showing with a reduction of at least one point in cardiovascular SOFA between *t*_0_ and *t*_72_ were considered “cardiovascular responders”; otherwise, they were “cardiovascular non-responders”.

Adverse events associated with PMX-HP were recorded and defined as tachycardia-heart rate (HR) >100 bpm or HR increase >10 % HR pre-treatment during 10′ from the beginning of PMX-HP, hypotension-MAP <70 mmHg or PAM reduction >10 % MAP pre-treatment during 10′ from the beginning of PMX-HP, bleeding-every type of hemorrhagic complication, cartridge clotting-coagulation of polymyxin-B fiber cartridge. Clotting of the PMX cartridge is an adverse event because it causes a premature interruption of the treatment (limiting its benefit) and blood lost for the patient.

IRBs and Ethical Committee of all centers approved data collection and the study.

### Statistical analysis

Normality tests were performed using the Kolmogorov–Smirnov test. Quantitative normally distributed variables (APACHE II, SAPS II, SOFA score and body temperature) were presented as mean ± standard deviation (SD) and non-normally distributed variables (age, time to treatment, vasopressors, EAA and biochemical data) as medians and the 25th–75th quartiles range. Univariate comparison was performed to compare variables between two groups. The organ dysfunction scores were compared using the unpaired *t* test. Categorical variables were expressed as actual numbers and percentages. In all comparisons, *p* < 0.05 was considered statistically significant.

Analysis was performed using R statistical software. Student’s *t* test, Wilcoxon test and Mann–Whitney test were used to compare means in case of normal paired or unpaired samples, non-normal paired samples and non-normal unpaired samples, respectively. Fisher’s exact test was carried out to evaluate significance in contingency tables. Multiple logistic regression was performed to evaluate the dependence of categorical variables on one or more predictors, and linear multiple regression was used to model the dependence of scalar variables on one or more explanatory variables. Cox proportional hazard model was used to relate time-to-death to multiple covariates. For all of the statistics tests and models, a 0.05 significance level was considered.

## Results

Demographic and clinical data of the 357 patients treated with PMX-HP are reported in Table 1. Data were recorded by 35 out of 57 centers with a median of patients per center of 9 [3–13] in Europe and 4.5 (2.7–11) in Asia.

**Table 1:** Demographic characteristics of patients treated with PMX-HP

Demographic characteristics	*N* 357
Age, years, mean ± SD	63 (51–72)
Gender, male	240 (67.2)
SAPS II at admission, mean ± SD	50.3 ± 19.2
APACHE II at admission, mean ± SD	21.8 ± 7.2
SOFA at admission, mean ± SD	10.9 ± 3.5
Incidence of shock	305 (85.4)
Source of sepsis	
Abdominal	157 (44)
Pulmonary	63 (17.6)
Urinary	16 (4.5)
Cardiac	23 (6.4)
Trauma	19 (5.3)
Other^a^	79 (22)
Patients without microbiological cultures *N* (%)	139 (38.9)
Patients with microbiological cultures	218 (61.1)
Negative microbiological cultures	41 (18.8)
Positive microbiological cultures	177 (81.1)
Gram positive only	17 (7.8)
Gram negative only	81 (37.2)
Fungi only	13 (6.0)
Mixed including gram negatives	51 (23.4)
Mixed not including gram negatives	8 (3.7)
Patients with endotoxin activity assay, *N* (%)	132 (37)
Abdominal	47 (35.6)
Non-abdominal	85 (64.4)
Without microbiology data	33 (25)
Negative cultures	12 (9)
Gram positive only	7 (5.3)
Gram negative only	43 (32.6)
Fungi only	14 (10.6)
Mixed including gram negatives	21 (15.9)
Mixed not including gram negatives	3 (2.2)
Patients with 2 treatments, *N* (%)	219 (61)
Patients with 1 treatment, *N* (%)	138 (39)
28 days survival	180 (54.5)
ICU survival	192 (55.2)
Hospital survival	172 (50)

Data are expressed as *N* (%) apart from otherwise indicated

^a^Other includes soft tissue infections, CVC-related infections, meningitides

Septic shock was diagnosed in 305 patients and severe sepsis in 52 patients. Eight patients had not data about vasopressor infused and they were considered presumably affected by severe sepsis. Main sources of infection were the abdomen (44 %) and the lung (17.6 %).

EA was recorded in 18 out of 24 centers in which the assay was available. EAA was measured in 132 out of 357 patients (37.0 %) with a median value of 0.77 (0.69–0.90) at *t*_0_. EAA was ≥0.6 in 120 patients and <0.6 in 12 patients, but the small number did not allowed any comparison between two groups.

Microbiological evidence of infection was documented in 174 (49.5 %) patients. Gram negative bacteria were isolated in 60.6 % of the microbiological samples (37.2 % mono-microbial and 23.4 % poly-microbial). In 139 patients, microbiological results were either not or partly reported.

Each session of PMX-HP theoretically lasted 2 h. No data about reason (except cartridge clotting) and timing of its premature interruption were recorded in the registry.

The majority of patients (73.8 %) were treated within 24 h from the onset of severe sepsis and septic shock; 13.2 % of patients received the PMX-HP between 24 and 48 h after the diagnosis of severe sepsis and septic shock, while 12.9 % of patients had a delay of more than 2 days.

### Safety of Polymyxin-B hemoperfusion

Two cycles of PMX-HP were performed in 219 patients; 138 patients received only one treatment.

Twenty-six adverse events were recorded (two tachycardias, ten hypotensions, one bleeding, thirteen cartridge clottings) (Additional file 1: Table S12).

Among the PMX-HP clotted cartridge, in eight patients was not administered heparin bolus at the beginning of the treatment and a lower amount of continuous heparin infusion was prescribed as compared to the recommended standard dose. No information about heparin dosage was reported for the remaining five clotted cartridges.

None of these adverse events caused clinical worsening or death of the patients.

### Effect of PMX-HP on organ dysfunction

SAPS II score was 48.0 ± 16.6 in patients suffering from severe sepsis versus 50.5 ± 19.5 in patients suffering from septic shock patients. SOFA score at *t*_0_ was 8.1 ± 2.9 in patients suffering from severe sepsis versus 12.9 ± 4.1 in patients suffering from septic shock patients; 72 h from the beginning of PMX-HP, SOFA score decreased in patients suffering from septic shock, but not in patients suffering from severe sepsis (10.7 ± 5.4 vs 8.9 ± 4.3, respectively).

Table 2 shows the variations of each SOFA component between *t*_0_ and *t*_72_.

**Table 2:** Variables changes 72 h after PMX-HP

Patients	*t*_0_ *N* = 357	*t*_72_ *N* = 299	*p* (Wilcoxon)
SOFA score	12.4 ± 4.2	10.5 ± 5.3	*<0.001*
Cardiovascular SOFA	3.32 ± 1.29	2.16 ± 1.77	*<0.001*
Renal SOFA	2.23 ± 1.62	1.84 ± 1.77	*0.013*
Hepatic SOFA	1.22 ± 1.28	1.19 ± 1.30	0.80
Respiratory SOFA	2.40 ± 1.06	1.95 ± 0.95	*<0.001*
Coagulation SOFA	1.33 ± 1.29	1.67 ± 1.38	*0.004*
Inotropic score	30 (11.9–72.5)	6.0 (0.0–22)	*<0.001*
Lactate, mmol/L	3.4 (1.9–6.0)	1.9 (1.3–2.9)	*<0.001*
Platelets, 10^3^/µL	117 (56–220)	86 (40–163)	*<0.001*

Normally distributed data are expressed as mean ± SD and non-normally distributed data as median (interquartile range)

Italics indicates significant *p* values

A significant improvement of cardiovascular (*t*_0_ = 3.32 ± 1.29 vs. *t*_72_ = 2.16 ± 1.77; *p* < 0.001), respiratory (*t*_0_ = 2.40 ± 1.06 vs. *t*_72_ = 1.95 ± 0.95; *p* < 0.001) and renal (*t*_0_ = 2.23 ± 1.62 vs. *t*_72_ = 1.84 ± 1.77; *p* = 0.013) scores with a reduction of inotropic score [*t*_0_ = 30.0 (11.9–72.5) vs. *t*_72_ = 6.0 (0.0–22.0); *p* < 0.001] and lactate levels [*t*_0_ = 3.4 (1.9–6.0) vs. *t*_72_ = 1.9 (1.3–2.9); *p* < 0.001] were observed after a follow-up of 72 h. Only the coagulation score significantly increased (*t*_0_ = 1.33 ± 1.29 vs. *t*_72_ = 1.67 ± 1.38; *p* = 0.004) together with platelet reduction [*t*_0_ = 117 (56–220) vs. *t*_72_ = 86 (40–163); *p* < 0.001]; both were not responsible of meaningful clinical worsening. These results were confirmed excluding patients dead at 72 h except for renal SOFA improvement (Additional file 1: Table S1).

### Length of stay and mortality

Median ICU and hospital length of stay (LoS) were 16 days (8–29) and 28 days (16–49), respectively. Overall 28-day survival after PMX-HP was 54.5 %. Patients with abdominal sepsis treated within 24 h from the diagnosis of septic shock had a survival rate of 64.5 %.

A Cox regression model, stratified by age, was carried out to identify the independent covariates affecting 28-day overall survival (Table 3). The variation of cardiovascular, respiratory and coagulation SOFA were identified as independent covariates for 28-day survival. No significant difference in survival was observed between patients receiving one or two treatments (*p* = 0.44).

**Table 3:** Cox regression on mortality stratified by age

	Univariate		*p*	Multivariate		*p*
	HR	95 % CI		HR	95 % CI	
*Septic source*						
Abdominal versus respiratory	1.56	0.93–2.63	0.09	0.99	0.25–3.97	0.99
Abdominal versus urinary	0.47	0.14–1.61	0.23	0.86	0.04–16.75	0.92
Cardiovascular SOFA at *t*_0_	1.17	0.98–1.41	0.09	1.61	0.98–2.67	0.06
Coagulation SOFA at *t*_0_	1.10	0.95–1.28	0.21	0.96	0.68–1.36	0.83
Respiratory SOFA at *t*_0_	1.14	0.94–1.39	0.17	1.42	0.83–2.44	0.20
Renal SOFA at *t*_0_	0.97	0.81–1.17	0.75	0.85	0.53–1.38	0.52
Liver SOFA at *t*_0_	1.17	0.98–1.39	0.08	1.38	0.89–2.14	0.15
Δ Cardiovascular SOFA	1.39	1.20–1.62	*<0.001*	1.68	1.21–2.32	*0.002*
Δ Coagulation SOFA	1.46	1.13–1.90	*0.004*	1.64	1.07–2.50	*0.022*
Δ Respiratory SOFA	1.26	1.01–1.56	*0.04*	1.68	0.99–2.84	*0.05*
Δ Renal SOFA	1.29	0.94–1.76	0.11	0.83	0.47–1.47	0.51
Δ Liver SOFA	1.74	1.20–2.52	0.003	1.66	0.93–2.94	0.08
Timing^a^	0.99	0.96–1.02	0.61	0.95	0.88–1.01	0.20
European versus asiatic	0.53	0.33–0.86	*0.001*	2.51	0.29–22.03	0.41

Italics indicates significant *p* values

^a^Time between the diagnosis of severe sepsis/septic shock and PMX-HP

Factors influencing the lack of cardiovascular response have been evaluated by a logistic regression (Table 4).

**Table 4:** Factors influencing the lack of cardiovascular response

All patients	Odds ratio	95 % CI	*p*
Age	0.99	0.97–1.02	0.82
Cardiovascular SOFA at *t*_0_	1.51	1.07–2.13	*<0.019*
Coagulative SOFA at *t*_0_	0.96	0.75–1.24	0.78
Liver SOFA at *t*_0_	1.09	0.83–1.42	0.54
Renal SOFA at *t*_0_	1.17	0.88–1.55	0.28
Respiratory SOFA at *t*_0_	0.54	0.39–0.75	*<0.001*
European versus Asian	1.38	0.43–4.42	0.59

Italics indicates significant *p* values

Cardiovascular and respiratory SOFA at *t*_0_ were independent factors influencing cardiovascular response. Cardiovascular response was positively associated with the former [OR 1.51 (1.07–2.13, 95 % CI with *p* < 0.019)] and negatively with the latter [OR 0.54 (0.39–0.75, 95 % CI with *p* < 0.001)].

Cardiovascular SOFA at baseline between responders and non-responders (3.7 ± 0.7 vs. 3.2 ± 1.4, *p* ≤ 0.001) was significantly different. Survival rates between cardiovascular responders and non-responders were 74.6 and 41.9 %, respectively (*p* < 0.001, Additional file 2: Figure S1). No significant differences in terms of age and SAPS II (48.6 ± 19.2 vs 51.2 ± 19.2, *p* = 0.32) were detected between the two subgroups (Additional file 1: Table S3).

The univariate Cox regression model identified the geographical origin as a factor influencing the mortality.

The characteristics of European and Asian patients are reported in Table 5.

**Table 5:** Characteristics of enrolled patients by geographical origin

Characteristics of enrolled patients by origin	*N* = 297	*N* = 60	*p*
	European	Asian	
Age, years	66 (54–74)	54 (41.3–62)	*<0.001*
SOFA score at admission	10.8 ± 3.5	12.3 ± 3.4	0.16
SAPS II at admission	49.6 ± 18.0	53.8 ± 25.2	0.21
Incidence of shock *N* (%)	257 (86.5)	48 (80)	0.31
Septic source *N* (%)			
Abdominal	142 (47.8)	15 (25)	*0.002*
Pulmonary	42 (14.1)	21 (35)	*<0.001*
Urinary	13 (4.4)	3 (5)	0.90
Postoperative cardiac surgery	22 (7.4)	1 (1.7)	0.17
Trauma	19 (6.4)	0	–
Other	59 (19.9)	20 (33.3)	*0.03*
Days from diagnosis to enrollment *N* (range)	1 (0–2.0)	1.0 (0.0–1.0)	0.45
SOFA score at enrollment	11.8 ± 3.9	15.6 ± 4.7	*<0.001*
Cardiovascular SOFA at *t*_0_	3.4 ± 1.2	3.2 ± 1.5	0.38
Renal SOFA at *t*_0_	2.2 ± 1.6	2.6 ± 1.6	0.08
Hepatic SOFA at *t*_0_	1.1 ± 1.2	1.9 ± 1.6	*<0.001*
Respiratory SOFA at *t*_0_	2.3 ± 1.0	2.8 ± 1.1	*0.002*
Coagulation SOFA at *t*_0_	1.3 ± 1.3	1.5 ± 1.3	0.17
Inotropic score at *t*_0_	6.0 (0.0–20.3)	5.0 (0.0–49.3)	0.15
Lactate at *t*_0_, mmol/L	1.9 (1.2–2.8)	2.7 (1.5–5.2)	0.18
Platelets at *t*_0_, 10^3^/µL	84 (35.3–166.8)	90 (55.0–129.0)	0.12
SOFA score at *t*_72_	10.2 ± 5.1	13.3 ± 6.4	*<0.001*
Cardiovascular SOFA at *t*_72_	2.2 ± 1.8	2.0 ± 1.9	0.64
Renal SOFA at *t*_72_	1.8 ± 1.8	2.2 ± 1.8	0.92
Hepatic SOFA at *t*_72_	1.1 ± 1.3	2.0 ± 1.5	*0.006*
Respiratory SOFA at *t*_72_	1.9 ± 0.9	2.0 ± 1.1	0.92
Coagulation SOFA at *t*_72_	1.7 ± 1.4	1.7 ± 1.2	0.72
Inotropic Score at *t*_72_	6.0 (0.0–20.3)	5.0 (0.0–49.3)	0.76
Lactates at *t*_72_, mmol/L	1.9 (1.2–2.8)	2.7 (1.5–5.2)	0.09
Platelets at *t*_72_, 10^3^/µL	84 (35.3–166.8)	90 (55.0–129.0)	0.63
28-day survival, *N* (%)	160 (58.8)	20 (34.5)	*0.003*
ICU survival, *N* (%)	170 (59)	22 (36.7)	*0.006*
Hospital survival, *N* (%)	158 (53.2)	21 (35)	*0.02*

Normally distributed data are expressed as mean ± SD and non-normally distributed data as median (interquartile range)

Italics indicates significant *p* values

Europeans were older [66 (54–74) years vs. 54 (41.3–62), *p* < 0.001], their SOFA score at enrollment was lower (11.8 ± 3.9 vs. 15.6 ± 4.7, *p* < 0.001), the length of ICU and hospital stay was shorter [ICU LoS 11 (1–123) days vs. 17 (1–213), *p* < 0.001; hospital LoS 14.5 (2–140) days vs. 34 (2–407), *p* < 0.001], and they had a higher survival rate at 28-day than Asian (Additional file 3: Figure S2), both in the ICU (59 vs. 36.7 %, *p* = 0.006) and in the hospital (53.2 vs. 35.0 %, *p* = 0.02).

The degree of cardiovascular response to PMX-HP treatment was similar between the two populations (47.4 % in European compared to 50 % in Asian patients), as well as the time between the diagnosis and start of the treatment.

## Discussion

The EUPHAS 2 data collection represents the largest database on PMX-HP application describing its utilization in the real clinical life. EUPHAS 2 registry was created to describe the actual use of the PMX-HP in the real life collecting data from unselected populations of septic patiens. PMX-HP is a complementary retreatment applied to patients affected by severe sepsis and septic shock that not respond to standard care.

The registry lacked of data about fluid load administered and hemodynamic monitor directing fluid resuscitation. However, each center performed PMX-HP only after the application of international consensus recommendation for sepsis, as strategy of resuscitation.

Surprisingly, in 142 patients (42 %) with clinical diagnosis of severe sepsis or septic shock, microbiological culture results were not reported that, if not performed, indicates a poor compliance to the bundles recommendations [20].

In 68 patients, EA was high although microbiological analysis did not reveal the presence of Gram negative germs. We can only suspect that the measured EA came from a Gram negative component of infection, which was either not revealed in microbiological tests or it was caused by direct translocation of endotoxin due to sepsis-induced permeability of the gut.

PMX-HP seemed to be safe: no major adverse events were reported. Data analysis showed a significant platelet count reduction at *t*_72_ without clinical worsening of the patients. The phenomenon observed could be a manifestation of the septic cascade evolution, which could alter platelet count and function, otherwise a complication of the extracorporeal technique. Data collected in the registry did not permit to establish a relationship between platelet decline and PMX-HP or other causes. The majority of patients who experienced a severe decline in platelet numbers were also receiving Renal Replacement Therapy.

Despite the retrospective nature of EUPHAS 2 data collection and the lack of a control group, the relevant number of patients allowed a reliable statistical analysis of PMX-HP feasibility in a real clinical context.

Recently published epidemiologic studies reported a 48.7 % in-hospital mortality for septic shock in France [26], as well as a 44 % hospital mortality for severe sepsis in Asian countries [27], having both the same period of time of EUPHAS 2. The comparison between EUPHAS 2 and epidemiologic studies in France [26] and Asia [27] showed a trend to clinical improvement without significant adverse event associated with endotoxin removal. Patients with abdominal infections treated within 24 h from the diagnosis of septic shock, had a 28-day survival of 64.5 %, which was comparable with 68 % observed in EUPHAS [15].

Recently, Zhou et al. [28] extended the analysis of effectiveness to all types of extracorporeal therapies in sepsis RCTs and concluded that the effect on mortality was mainly driven by studies using PMX-HP.

Iwagami et al. [29] published a retrospective analysis on 590 patients treated with PMX-HP matched through a propensity score to 590 patients treated with standard care who had open abdominal surgery on the day of admission for perforation of lower gastrointestinal tract, requiring the infusion of vasopressors. The authors showed a 28-day mortality of 17.1 % (101 of 590) in the PMX-HP group and 16.3 % (96 of 590) in the control group (*p* = 0.696). Data came from a nationwide inpatient database in Japan, showing no benefit of PMX-HP in a less severe population characterized by a low mortality rate. However, Payen et al. in the ABDOMIX randomized controlled trial [16] observed an absence of benefit from the use of PMX membranes to treat peritonitis-induced septic shock after emergency surgery.

In ABDOMIX, the 28-day mortality rate was close to recent clinical RCTs on selected patient population affected by septic shock [30] unlike EUPHAS 2, which is a registry without specific inclusion criteria. ABDOMIX excluded patients affected by life-threatening abdominal diseases included in EUPHAS 2 as ischemia, obstruction, inflammatory colitis and trauma. In ABDOMIX, only 81 over the 119 treated patients had completed two sessions of PMX-HP unlike EUPHAS [15], in which all patients enrolled had completed the two PMX-HP planned with a definitely higher mortality in the control group. In ABDOMIX, the isolation of yeasts from peritoneum was more frequent in PMX-treated group than in controls: this kind of disease represents a severe life-threatening condition not susceptible to improve by the endotoxin-adsorption blood purification techniques.

EUPHAS 2 confirmed that PMX-HP is often used to treat non-abdominal infection. The respiratory source was in fact the second clinical condition most frequently observed in the study and in this context the efficacy of the PMX-HP seemed less efficient. ICU and hospital survival were quite similar between patients with abdominal and respiratory sepsis, but patients with respiratory infections showed a trend toward a higher mortality rate at 28 days (Additional file 1: Table S2).

Lower respiratory tract infections might have some differences compared to abdominal or urinary sepsis. Pulmonary sepsis seems to respond less to the two sessions of endotoxin removal because source control is substantially achieved through the antibiotic action and the resolution needs a longer time. Differently in patients with intra-abdominal sepsis, the source control is obtained through surgery and the concomitant PMX treatment, achieving a more rapid resolution.

Another limitation of EUPHAS 2 is the lack of information about patients comorbidities and early antibiotic therapy appropriately applied, which cannot be obtained from the phase 1 of the registry. The phase 2 will clarify the implication of these important factors related to the outcome.

The database included both 297 European and 60 Asian patients. Even if baseline characteristics of patients at admission were similar between the two groups except for the age (Asian were younger than European, respectively 51.6 ± 14.7 vs 62.1 ± 14.8 years, *p* < 0.001), there was a significant difference at the enrollment to PMX-HP in terms of SOFA score and septic source. European and Asiatic population were not comparable for the septic source identified: Abdominal sepsis was prevalent in European patients, and pulmonary sepsis was most frequent in Asian patients.

Asiatic patients had a SOFA score higher than European patients (15.6 ± 4.7 vs 12.4 ± 4.2 respectively, *p* < 0.001), especially for respiratory and hepatic components. Phua et al. [27] reported a lower compliance with surviving sepsis campaign bundles in Asian ICUs, particularly during the first 24 h. A lower adherence to bundles correlated with worsening clinical condition and higher mortality.

Both European and Asian patients showed a similar clinical effect of PMX-HP treatment on organ dysfunction, that is mainly a significant reduction of vasopressor dependence and an improvement in gas exchange.

Published papers have underlined the positive effect of endotoxin removal by PMX-HP on cardiovascular dysfunction both increasing the arterial pressure and reducing the need of vasopressors.

In EUPHAS 2 registry, the cardiovascular component of SOFA represented 26.7 % of the overall score when the treatment was started. This means that enrolled patients were likely to have a high vasopressor load. Several studies showed that a higher vasopressor load is related to a higher risk of death in septic shock. A recent publication by Monti et al. [31] analyzed the response to PMX-HP of patients affected by refractory septic shock. They concluded that an early hemodynamic improvement highlighted by a vasopressor load reduction, potentially attenuates the mortality rate. The Cox regression analysis of 28-day mortality recognized the variation between baseline and 72 h of cardiovascular and coagulation components of SOFA score as independent covariates.

The similar degree of cardiovascular response to the PMX-HP treatment did not reflect a similar effect on mortality in Europe and Asia (Additional file 4: Figure S3, Additional file 5: Figure S4). Asian patients showed a lower survival rate and a higher length of stay than European patients, both justified by the worse organ dysfunction at the beginning of PMX-HP despite a cardiovascular response similar to European patients. The worse outcome of Asian patients could be related to the lower adherence to sepsis guideline and the higher incidence of respiratory infection than European patients.

In conclusion, notwithstanding the retrospective nature of the present data collection and the lack of a control group, these data confirmed the feasibility of the PMX-HP application in the usual clinical context without life-threatening adverse events related to its application, as already described in previous RCTs and observational studies. The ongoing EUPHRATES RCT [32] will clarify the effective clinical collocation of PMX-HP in patients suffering from endotoxin-mediated septic shock.

## Additional files


10.1186/s13613-016-0178-9 Supplementary Material.

10.1186/s13613-016-0178-9 Kaplan-Meier cardiovascular responders vs cardiovascular non responders.

10.1186/s13613-016-0178-9 Kaplan-Meier European patients versus Asian patients.

10.1186/s13613-016-0178-9 Kaplan-Meier plot for European “responders/non-responders”.

10.1186/s13613-016-0178-9 Kaplan-Meier plot for Asian “responders/non-responders”.

## Abbreviations

EAA: endotoxin activity assay
EA: endotoxin activity
ICU: intensive care units
LPS: lipopolysaccharide
MOF: multiple organ failure
PMX-HP: polymyxin-B hemoperfusion
RCT: randomized controlled trial
SS: severe sepsis
SSh: septic shock
HR: heart rate
MAP: mean arterial pressure
