# Supplementary material for: Polymyxin-B hemoperfusion in septic patients: analysis of a multicenter registry
Source: Ann Intensive Care. 2016 Aug 8;6:77. doi: 10.1186/s13613-016-0178-9 (PMC4977232; doi:10.1186/s13613-016-0178-9)
Supplement: Supplementary file 1 — 10.1186/s13613-016-0178-9 Supplementary Material. [file 13613_2016_178_MOESM1_ESM.docx]

**Electronic Supplementary Material**

**TABLE S1. Variables changes 72 hours after PMX-HP for patients alive at 72 hours**

| **Patients Survivors**  **at 72 hours (N=299)** | **t0** | **t72** | **P (Wilcoxon)** |
| --- | --- | --- | --- |
| **SOFA score** | 11.7± 4.0 | 10.2± 5.1 | **0.001** |
| **Cardiovascular SOFA** | 3.2 ±1.4 | 2.1±1.8 | **<0.001** |
| **Renal SOFA** | 2.1±1.6 | 1.8±1.8 | 0.07 |
| **Hepatic SOFA** | 1.1±1.2 | 1.2±1.3 | 0.94 |
| **Respiratory SOFA** | 2.4±1.0 | 1.9±1.0 | **<0.001** |
| **Coagulation SOFA** | 1.3±1.3 | 1.6±1.4 | **0.01** |
| **Inotropic Score** | 29.1(10.0-68.5) | 6.0(0.0-20.5) | **<0.001** |
| **Lactates, mmol/L** | 3.0(1.8-5.7) | 1.9(1.3-2.9) | **<0.001** |
| **Platelets, 10**^3^ **/µl** | 121(58-230) | 90(48.5-163.5) | **<0.05** |

***Normally distributed data are expressed as mean± SD and –non-normally distributed data as median (interquartile range).**

**TABLE S2. Overall abdominal and pulmonary survival rate**

|  | **Abdominal** | **Pulmonary** | **p** |
| --- | --- | --- | --- |
| **28-survival N(%)** | **86/142 (60.6)** | **29/61 (47.5)** | **0.13** |
| **ICU survival N(%)** | **89/152 (58.6)** | **30/62 (48.4)** | **0.23** |
| **Hospital survival N(%)** | **81/152 (53.3)** | **29/62 (46.8)** | **0.43** |

**TABLE S3. Baseline characteristics among cardiovascular responders and non-responders**

| **All patients** | **Responders** | **Non-Responders** | **p** |
| --- | --- | --- | --- |
| **Age, years** | 66(55-73) | 63(51-72) | 0.21 |
| **SAPSII score** | 48.6±19.27 | 51.20±19.25 | 0.32 |
| **SOFA score at admission** | 11.71±3.92 | 12.68±4.05 | 0.92 |
| **Cardiovascular SOFA at t0** | 3.7±0.7 | 3.2±1.4 | **<0.001** |
| **Coagulative SOFA at t0** | 1.3±1.3 | 1.4±1.3 | 0.36 |
| **Liver SOFA at t0** | 1.2±1.1 | 1.1±1.3 | 0.50 |
| **Renal SOFA at t0** | 1.7±1.4 | 1.5±1.5 | 0.30 |
| **Respiratory SOFA at t0** | 2.0±1.0 | 2.4±1.0 | **0.001** |

***Normally distributed data are expressed as mean± SD and –non-normally distributed data as median (interquartile range).**

**TABLE S4. European patients characteristics**

| **European** | **t0** | **t72** | **P (Wilcoxon)** |
| --- | --- | --- | --- |
| **SOFA score** | 11.8±3.9 | 10.2±5.1 | **0.001** |
| **Cardiovascular SOFA** | 3.4±1.2 | 2.2±1.8 | **<0.001** |
| **Renal SOFA** | 2.2±1.6 | 1.8±1.8 | **0.02** |
| **Hepatic SOFA** | 1.1±1.2 | 1.1±1.3 | 0.92 |
| **Respiratory SOFA** | 2.3±1.0 | 1.9±0.9 | **<0.001** |
| **Coagulation SOFA** | 1.3±1.3 | 1.7±1.4 | **0.004** |
| **Inotropic Score** | 30(11.9-70) | 6.0(0.0-20.3) | **<0.001** |
| **Lactates, mmol/l** | 3.4 (1.9-5.8) | 1.9(1.2-2.8) | **<0.001** |
| **Platelets,**10^3^/µl | 121(55.5-2.31) | 84(35.3-166.8) | **0.001** |

***Normally distributed data are expressed as mean± SD and –non-normally distributed data as median (interquartile range).**

**TABLE S5. Asian patients characteristics**

| **Asian** | **t0** | **t72** | **P (Wilcoxon)** |
| --- | --- | --- | --- |
| **SOFA score** | 15.6±4.7 | 13.3±6.4 | 0.15 |
| **Cardiovascular SOFA** | 3.12±1.5 | 2.0±1.9 | **0.001** |
| **Renal SOFA** | 2.6±1.6 | 2.2±1.8 | 0.29 |
| **Hepatic SOFA** | 1.9±1.6 | 2.0±1.5 | 0.95 |
| **Respiratory SOFA** | 2.8±1.1 | 2.0±1.1 | **0.001** |
| **Coagulation SOFA** | 1.5±1.3 | 1.7±1.2 | 0.46 |
| **Inotropic Score** | 42.5(13.8-82) | 5.0(0.0-49.3) | 0.06 |
| **Lactates, mmol/l** | 4.4(1.0-8.8) | 2.7(1.5-5.2) | 0.19 |
| **Platelets,** 10^3^/µl | 90(58.0-164.0) | 90.0(55.0-129.0) | 0.75 |

***Normally distributed data are expressed as mean± SD and –non-normally distributed data as median (interquartile range).**

**TABLE S6. Characteristics of Abdominal patients by origin**

|  | **Origin** | | **P** |
| --- | --- | --- | --- |
|  | **European**  **N=142** | **Asian**  **N=15** |  |
| **Age, years** | 68.0(57.3-75.8) | 50.0(37.8-58.8) | **<0.001** |
| **SOFA score at admission** | 11.2±3.3 | 12.3±3.4 | 0.09 |
| **SAPS II at admission** | 49.1±19.6 | 69.3±21.8 | **0.01** |
| **Incidence of shock N(%)** | 128 (90.1) | 15 (86.7) | 0.86 |
| **Days from diagnosis to enrolment N [range]** | 1.0 [0.0-1.0] | 0,0[0.0-1] | 0.19 |
| **SOFA score at enrollment** | 11.1±3.8 | 17.5±3.1 | **<0.001** |
| **Cardiovascular SOFA at t0** | 3.4±1.1 | 3.5±1.4 | 0.92 |
| **Renal SOFA at t0** | 2.0±1.7 | 2.7±1.7 | 0.17 |
| **Hepatic SOFA at t0** | 1.0±1.2 | 2.2±1.3 | **0.02** |
| **Respiratory SOFA at t0** | 2.4±1.0 | 2.8±1.1 | 0.26 |
| **Coagulation SOFA at t0** | 1.0±1.2 | 1.8±1.1 | **0.01** |
| **Inotropic Score at t0** | 28.5(12.0-59.7) | 70.0(35.0-85.0) | **0.04** |
| **Lactates at t0, mmol/l** | 3.5(2.1-6.4) | 8.5(4.8-13.6) | **0.01** |
| **Platelets at t0, 10^3^/µl** | 157.0(90.7-253.2) | 80.0(50.5-103.0) | **0.01** |
| **Cardiovascular SOFA at t72** | 2.0±1.7 | 1.7±2.0 | 0.64 |
| **Renal SOFA at t72** | 1.5±1.7 | 2.5±1.9 | 0.13 |
| **Hepatic SOFA at t72** | 0.9±1.1 | 2.0±1.2 | **0.02** |
| **Respiratory SOFA at t72** | 2.0±0.9 | 2.1±1.4 | 0.81 |
| **Coagulation SOFA at t72** | 1.4±1.4 | 1.6±1.2 | 0.55 |
| **Inotropic Score at t72** | 5.0(0.0-17.0) | 0.0 (0.0-53.8) | 0.94 |
| **Lactates at t72, mmol/l** | 1.8(1.2-2.7) | 6.4 (4.9-10.8) | **<0.001** |
| **Platelets at t72, 10^3^/µl** | 118.5(54.7-193.2) | 99.0 (50.5-131.0) | 0.60 |
| **28-day survival (%)** | 62.2 | 46.7 | 0.31 |
| **ICU survival (%)** | 60.6 | 40.0 | 0.19 |
| **Hospital survival (%)** | 55.5 | 33.3 | 0.16 |

***Normally distributed data are expressed as mean± SD and –non-normally distributed data as median (interquartile range).**

**TABLE S7. European Abdominal Patients Characteristics**

| **European Abdominal Patients** | **t0** | **t72** | **P** |
| --- | --- | --- | --- |
| **SOFA score** | 11.1±3.8 | 9.0±5.2 | **0.001** |
| **Cardiovascular SOFA** | 3.4±1.1 | 2.0±1.7 | **<0.001** |
| **Coagulative SOFA** | 1.0±1.2 | 1.4±1.4 | **0.02** |
| **Liver SOFA** | 1.0±1.2 | 0.9±1.1 | 0.40 |
| **Renal SOFA** | 2.0±1.7 | 1.5±1.7 | **0.02** |
| **Respiratory SOFA** | 2.4±1.0 | 2.0±0.9 | **<0.001** |
| **Inotropic Score** | 28.5(12.0-59.7) | 5.0(0.0-17.0) | **<0.001** |
| **Lactates, mmol/l** | 3.5(2.1-6.4) | 1.8(1.2-2.7) | **<0.001** |
| **Platelets, 10^3^/µl** | 157.0(90.7-253.2) | 118.5(54.7-193.2) | **0.007** |

***Normally distributed data are expressed as mean± SD and –non-normally distributed data as median (interquartile range).**

**TABLE S8. Asian Abdominal Patients Characteristics**

| **Asian Abdominal Patients** | **t0** | **t72** | **P** |
| --- | --- | --- | --- |
| **SOFA score** | 17.5±3.1 | 12.1±8.1 | 0.09 |
| **Cardiovascular SOFA** | 3.5±1.4 | 1.7±2.0 | **0.02** |
| **Coagulative SOFA** | 1.8±1.1 | 1.6±1.2 | 0.62 |
| **Liver SOFA** | 2.2±1.3 | 2.0±1.2 | 0.73 |
| **Renal SOFA** | 2.7±1.7 | 2.5±1.9 | 0.77 |
| **Respiratory SOFA** | 2.8±1.1 | 2.1±1.4 | 0.21 |
| **Inotropic Score** | 70.0(35.0-85.0) | 0.0 (0.0-53.8) | **0.04** |
| **Lactates, mmol/l** | 8.5(4.8-13.6) | 6.4 (4.9-10.8) | 1.0 |
| **Platelets, 10^3^/µl** | 80.0(50.5-103.0) | 99.0 (50.5-131.0) | 0.35 |

***Normally distributed data are expressed as mean± SD and –non-normally distributed data as median (interquartile range).**

**TABLE S9. Characteristics of Pulmonary patients by origin**

|  | **Origin** | | **P** |
| --- | --- | --- | --- |
|  | **European**  **N=42** | **Asian**  **N=21** |  |
| **Age, years** | 61.5(42.5-69.8) | 54.5(50.0-59.0) | 0.21 |
| **SOFA score at admission** | 9.7±4.1 | 12.3±3.2 | 0.11 |
| **SAPS II at admission** | 49.4±16.2 | 54.5±18.8 | 0.37 |
| **Incidence of shock N(%)** | 37 (88.1) | 17 (81.0) | 0.44 |
| **Days from diagnosis to enrolment N [range]** | 2 [0-2.5] | 1.0 [0.0-2.0] | 0.35 |
| **SOFA score at enrollment** | 11.7±4.2 | 16.4±5.6 | **0.01** |
| **Cardiovascular SOFA at t0** | 3.5±1.2 | 3.4±1.3 | 0.73 |
| **Renal SOFA at t0** | 2.1±1.5 | 2.4±1.7 | 0.63 |
| **Hepatic SOFA at t0** | 0.8±0.9 | 1.8±1.8 | **0.03** |
| **Respiratory SOFA at t0** | 2.3±1.1 | 3.1±1.0 | **0.01** |
| **Coagulation SOFA at t0** | 1.3±1.3 | 1.4±1.3 | 0.91 |
| **Inotropic Score at t0** | 50.0(15.7-89.6) | 40.0(10.0-80.0) | 0.55 |
| **Lactates at t0, mmol/l** | 3.4(1.9-5.5) | 4.0(1.4-6.9) | 0.75 |
| **Platelets at t0, 10^3^/µl** | 123.0(49.0-249.5) | 91.0(60.0-184.0) | 0.83 |
| **Cardiovascular SOFA at t72** | 2.5±1.8 | 3.3±1.0 | 0.13 |
| **Renal SOFA at t72** | 2.5±1.8 | 1.1±1.8 | **0.01** |
| **Hepatic SOFA at t72** | 0.9±1.0 | 1.9±1.9 | 0.21 |
| **Respiratory SOFA at t72** | 2.1±1.0 | 2.0±0.9 | 0.73 |
| **Coagulation SOFA at t72** | 1.5±1.5 | 2.1±1.2 | 0.27 |
| **Inotropic Score at t72** | 10.0(0.0-58.0) | 17.5(5.5-27.3) | 0.44 |
| **Lactates at t72, mmol/l** | 2.1(1.5-3.2) | 2.5(1.3-3.2) | 0.71 |
| **Platelets at t72, 10^3^/µl** | 87.0(54.0-217.7) | 64.0(10.0-30.0 | 0.21 |
| **28-day survival (%)** | 58.5 | 25 | **0.03** |
| **ICU survival (%)** | 58.5 | 28.6 | **0.05** |
| **Hospital survival (%)** | 56.1 | 28.6 | 0.07 |

***Normally distributed data are expressed as mean± SD and –non-normally distributed data as median (interquartile range).**

**TABLE S10. European Pulmonary Patients Characteristics**

| **European Pulmonary Patients** | **t0** | **t72** | **p** |
| --- | --- | --- | --- |
| **SOFA score** | 11.7±4.2 | 11.2±4.1 | 0.65 |
| **Cardiovascular SOFA** | 3.5±1.2 | 2.5±1.8 | **0.005** |
| **Coagulative SOFA** | 1.3±1.3 | 1.5±1.5 | 0.56 |
| **Liver SOFA** | 0.9±1.0 | 0.9±1.0 | 0.73 |
| **Renal SOFA** | 2.1±1.5 | 2.5±1.8 | 0.42 |
| **Respiratory SOFA** | 2.3±1.1 | 2.1±1.0 | 0.57 |
| **Inotropic Score** | 50.0(15.7-89.6) | 10.0(0.0-58.0) | 0.067 |
| **Lactates, mmol/l** | 3.4(1.9-5.5) | 2.1(1.5-3.2) | **0.017** |
| **Platelets, 10^3^/µl** | 123.0(49.0-249.5) | 87.0(54.0-217.7) | 0.68 |

***Normally distributed data are expressed as mean± SD and –non-normally distributed data as median (interquartile range).**

**TABLE S11. Asian Pulmonary Patients Characteristics**

| **Asian Pulmonary Patients** | **t0** | **t72** | **p** |
| --- | --- | --- | --- |
| **SOFA score** | 16.4±5.6 | 14. 5±3.0 | 0.35 |
| **Cardiovascular SOFA** | 3.4±1.3 | 3.3±1.0 | 0.80 |
| **Coagulative SOFA** | 1.4±1.3 | 2.1±1.2 | 0.15 |
| **Liver SOFA** | 1.8±1.8 | 1.9±1.9 | 0.96 |
| **Renal SOFA** | 2.4±1.7 | 1.1±1.8 | 0.09 |
| **Respiratory SOFA** | 3.1±1.0 | 2.0±0.9 | **0.01** |
| **Inotropic Score** | 40.0(10.0-80.0) | 17.5(5.5-27.3) | 0.49 |
| **Lactates, mmol/l** | 4.0(1.4-6.9) | 2.5(1.3-3.2) | 0.25 |
| **Platelets, 10^3^/µl** | 91.0(60.0-184.0) | 64.0(10.0-30.0 | 0.05 |

***Normally distributed data are expressed as mean± SD and –non-normally distributed data as median (interquartile range).**

**TABLE S12. ADVERSE EVENTS**

|  | **N** |
| --- | --- |
| **Treatments** | 576 |
| **Adverse events (1st treatment)** | 20 |
| **Tachycardia *** | 2 |
| **Hypotension **** | 8 |
| **Bleeding ***** | 1 |
| **Clotting ****** | 9 |
| **Adverse events (2nd treatment)** | 6 |
| **Tachycardia *** | 0 |
| **Hypotension**** | 2 |
| **Bleeding ***** | 0 |
| **Clotting ****** | 4 |

***Tachycardia: HR>100 bpm or HR increase>10% HR pre-treatment during 10’ from the beginning of PMX-DHP**

****Hypotension: MAP<70 mmHg or PAM reduction > 10% MAP pre-treatment during 10’ from the beginning of PMX-DHP or**

*****Bleeding: every type of hemorrhage complication**

****** Clotting: coagulation of Polymyxin B fiber cartridge which caused PMX-HP treatment interruption**
