# Supplementary material for: Polymyxin-B hemoperfusion in septic patients: analysis of a multicenter registry
Source: Ann Intensive Care. 2016 Aug 8;6:77. doi: 10.1186/s13613-016-0178-9 (PMC4977232; doi:10.1186/s13613-016-0178-9)
Supplement: Supplementary file 2 — 10.1186/s13613-016-0178-9 Kaplan-Meier cardiovascular responders vs cardiovascular non responders. [file 13613_2016_178_MOESM2_ESM.docx]

**FIGURE S1. Kaplan-Meier cardiovascular responders vs cardiovascular non responders**

P<0.001
