# Supplementary material for: Polymyxin-B hemoperfusion in septic patients: analysis of a multicenter registry
Source: Ann Intensive Care. 2016 Aug 8;6:77. doi: 10.1186/s13613-016-0178-9 (PMC4977232; doi:10.1186/s13613-016-0178-9)
Supplement: Supplementary file 4 — 10.1186/s13613-016-0178-9 Kaplan-Meier plot for European “responders/non-responders”. [file 13613_2016_178_MOESM4_ESM.docx]

**FIGURE S3. Kaplan-Meier plot for European “responders / non-responders”**

P<0.001
